# [4-(2-*tert*-But­oxy-2-oxoeth­oxy)naph­thalen-1-yl]diphenyl­sulfonium trifluoro­methane­sulfonate

**DOI:** 10.1107/S1600536812022167

**Published:** 2012-05-19

**Authors:** Sung Kwon Kang, Siyoung Jang

**Affiliations:** a Department of Chemistry, Chungnam National University, Daejeon 305-764, Republic of Korea

## Abstract

In the cation of the title salt, C_28_H_27_O_3_S^+^·CF_3_O_3_S^−^, the dihedral angle between the naphthalene ring system and the –C(=O)—O– plane is 80.39 (9)°. The three methyl groups of the *tert*-butyl group are each disordered over two orientations with an occupancy ratio of 0.712 (18):0.288 (18).

## Related literature

For the reactivity of sulfonium trifluoro­methane­sulfonate derivatives, see: McGarrigle *et al.* (2011 ▶); Scalfani & Bailey (2011 ▶); Yoshida (2012 ▶); Zhang *et al.* (2011 ▶).
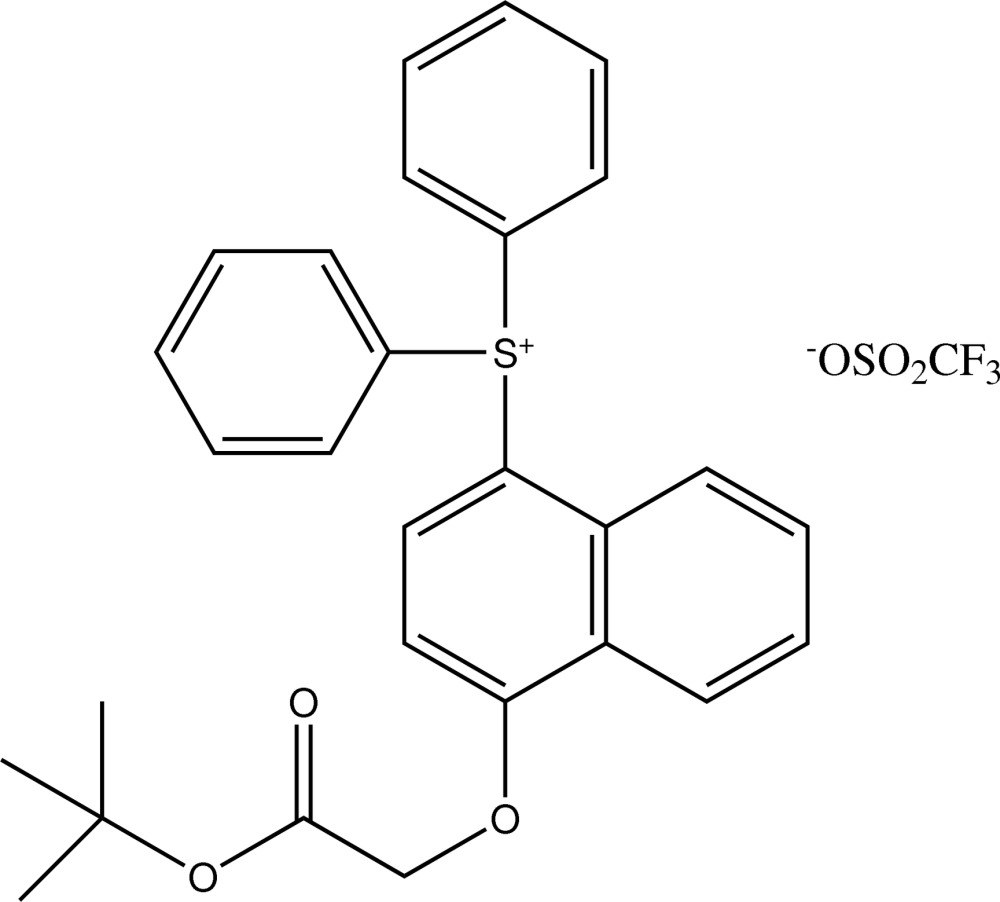


## Experimental

### 

#### Crystal data

C_28_H_27_O_3_S^+^·CF_3_O_3_S^−^
*M* *_r_* = 592.63
Monoclinic, 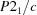

*a* = 10.8944 (2) Å
*b* = 17.2170 (4) Å
*c* = 15.6759 (2) Å
β = 102.834 (1)°
*V* = 2866.85 (9) Å^3^
*Z* = 4
Mo *K*α radiation
μ = 0.25 mm^−1^
*T* = 296 K
0.2 × 0.2 × 0.18 mm

#### Data collection

Bruker SMART CCD area-detector diffractometer
Absorption correction: multi-scan (*SADABS*; Bruker, 2002 ▶) *T*_min_ = 0.948, *T*_max_ = 0.955
54159 measured reflections
7117 independent reflections
4143 reflections with *I* > 2σ(*I*)
*R*_int_ = 0.053

#### Refinement

*R*[*F* ^2^ > 2σ(*F* ^2^)] = 0.061
*wR*(*F* ^2^) = 0.214
*S* = 0.99
7117 reflections
386 parameters
2 restraints
H-atom parameters constrained
Δρ_max_ = 1.00 e Å^−3^
Δρ_min_ = −0.52 e Å^−3^

### 

Data collection: *SMART* (Bruker, 2002 ▶); cell refinement: *SAINT* (Bruker, 2002 ▶); data reduction: *SAINT*; program(s) used to solve structure: *SHELXS97* (Sheldrick, 2008 ▶); program(s) used to refine structure: *SHELXL97* (Sheldrick, 2008 ▶); molecular graphics: *ORTEP-3 for Windows* (Farrugia, 1997 ▶); software used to prepare material for publication: *WinGX* (Farrugia, 1999 ▶).

## Supplementary Material

Crystal structure: contains datablock(s) global, I. DOI: 10.1107/S1600536812022167/tk5097sup1.cif

Structure factors: contains datablock(s) I. DOI: 10.1107/S1600536812022167/tk5097Isup2.hkl

Supplementary material file. DOI: 10.1107/S1600536812022167/tk5097Isup3.cml

Additional supplementary materials:  crystallographic information; 3D view; checkCIF report

## Figures and Tables

**Fig. 1.**
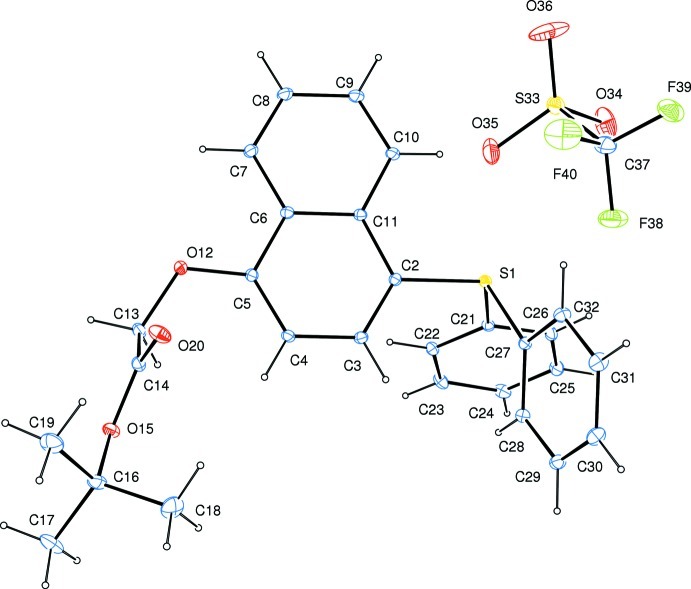
Molecular structure of the title compound, showing the atom-numbering scheme and 30% probability ellipsoids. Only major components of the disordered methyl groups are shown.

## supplementary crystallographic information

### Comment

The derivatives of sulfonium (trifluoromethanesulfonate) salts are interesting for their applications in various fields such as polymerization (Scalfani & Bailey, 2011; Yoshida, 2012) and syntheses of organic compounds (McGarrigle *et al.*, 2011; Zhang *et al.*, 2011). During the studies of these salts, we obtained crystals of the title compound.

The naphthalene unit is planar, with an r.m.s. deviation of 0.006 Å from the corresponding least-squares plane defined by the ten constituent atoms (C2 – C11). The bond distance of C14—O20 [1.1876 (34) Å] is much shorter than that of C14—C15 [1.3165 (31)], which is consistent with double bond character. In the cation, the naphthalene ring and ester group (C14—C16, O20) are almost perpendicular to each other, with a dihedral angle of 80.39 (9) °.

### Experimental

Phenyl sulfoxide (1.0 g, 5.0 mmol) and *tert*-butyl 2-(naphthalene-1-yloxy)acetate (1.29 g, 5.0 mmol) were dissolved in dichloromethane (50 ml) with stirring. Trifluoromethanesulfonic anhydride (1.41 g, 5.0 mmol) was slowly added to the above solution. The reaction solution was stirred for 30 minutes. The reaction mixture was washed with distilled water and after removing solvent with vacuum drying, a viscous oil was obtained. The oil was dissolved in dichloromethane and ether was slowly added. The white solid remained after the solvent was distilled off. Colourless crystals of (I) were obtained from its methanol solution by slow evaporation of the solvent at room temperature.

### Refinement

All the H atoms were positioned geometrically and refined using a riding model with C—H = 0.93–0.97 Å, and with *U*_iso_(H) = 1.2*U*_eq_(carrier C) for aromatic and methylene, and 1.5*U*_eq_(carrier C) for methyl-H atoms. The methyl groups on *t*-butyl in the cation are disordered with an occupancy ratio of 0.712 (18): 0.288 (18). In this disordered group, bond length restraints of C16—C17 and C16—C17A = 1.50 (2) Å were employed.

### Crystal data

**Table:**

C_28_H_27_O_3_S^+^·CF_3_O_3_S^−^	*F*(000) = 1232
*M**_r_* = 592.63	*D*_x_ = 1.373 Mg m^−^^3^
Monoclinic, *P*2_1_/*c*	Mo *K*α radiation, λ = 0.71073 Å
Hall symbol: -P 2ybc	Cell parameters from 8179 reflections
*a* = 10.8944 (2) Å	θ = 2.3–21.0°
*b* = 17.2170 (4) Å	µ = 0.25 mm^−^^1^
*c* = 15.6759 (2) Å	*T* = 296 K
β = 102.834 (1)°	Block, colourless
*V* = 2866.85 (9) Å^3^	0.2 × 0.2 × 0.18 mm
*Z* = 4	

### Data collection

**Table:**

Bruker SMART CCD area-detector diffractometer	4143 reflections with *I* > 2σ(*I*)
Graphite monochromator	*R*_int_ = 0.053
φ and ω scans	θ_max_ = 28.3°, θ_min_ = 1.8°
Absorption correction: multi-scan (*SADABS*; Bruker, 2002)	*h* = −14→14
*T*_min_ = 0.948, *T*_max_ = 0.955	*k* = −22→22
54159 measured reflections	*l* = −20→20
7117 independent reflections	

### Refinement

**Table:**

Refinement on *F*^2^	Primary atom site location: structure-invariant direct methods
Least-squares matrix: full	Secondary atom site location: difference Fourier map
*R*[*F*^2^ > 2σ(*F*^2^)] = 0.061	Hydrogen site location: inferred from neighbouring sites
*wR*(*F*^2^) = 0.214	H-atom parameters constrained
*S* = 0.99	*w* = 1/[σ^2^(*F*_o_^2^) + (0.1305*P*)^2^] where *P* = (*F*_o_^2^ + 2*F*_c_^2^)/3
7117 reflections	(Δ/σ)_max_ < 0.001
386 parameters	Δρ_max_ = 1.00 e Å^−^^3^
2 restraints	Δρ_min_ = −0.52 e Å^−^^3^

### Special details

**Table:**

Geometry. All s.u.'s (except the s.u. in the dihedral angle between two l.s. planes) are estimated using the full covariance matrix. The cell s.u.'s are taken into account individually in the estimation of s.u.'s in distances, angles and torsion angles; correlations between s.u.'s in cell parameters are only used when they are defined by crystal symmetry. An approximate (isotropic) treatment of cell s.u.'s is used for estimating s.u.'s involving l.s. planes.
Refinement. Refinement of *F*^2^ against ALL reflections. The weighted *R*-factor *wR* and goodness of fit *S* are based on *F*^2^, conventional *R*-factors *R* are based on *F*, with *F* set to zero for negative *F*^2^. The threshold expression of *F*^2^ > 2σ(*F*^2^) is used only for calculating *R*-factors(gt) *etc*. and is not relevant to the choice of reflections for refinement. *R*-factors based on *F*^2^ are statistically about twice as large as those based on *F*, and *R*- factors based on ALL data will be even larger.

### Fractional atomic coordinates and isotropic or equivalent isotropic displacement parameters (Å^2^)

**Table:**

	*x*	*y*	*z*	*U*_iso_*/*U*_eq_	Occ. (<1)
S1	0.51134 (6)	0.58122 (4)	0.25392 (4)	0.0433 (2)	
C2	0.5691 (2)	0.48539 (13)	0.24627 (16)	0.0423 (5)	
C3	0.6624 (2)	0.45595 (15)	0.30911 (16)	0.0459 (6)	
H3	0.6951	0.4852	0.3588	0.055*	
C4	0.7102 (2)	0.38210 (14)	0.30021 (16)	0.0470 (6)	
H4	0.7738	0.3621	0.3444	0.056*	
C5	0.6643 (2)	0.33901 (14)	0.22702 (16)	0.0420 (5)	
C6	0.5652 (2)	0.36839 (14)	0.15857 (15)	0.0421 (5)	
C7	0.5173 (3)	0.32518 (16)	0.08237 (17)	0.0541 (7)	
H7	0.5494	0.276	0.076	0.065*	
C8	0.4246 (3)	0.35455 (18)	0.01797 (19)	0.0667 (8)	
H8	0.3942	0.3256	−0.0323	0.08*	
C9	0.3747 (3)	0.42793 (18)	0.02682 (19)	0.0670 (9)	
H9	0.31	0.447	−0.0172	0.08*	
C10	0.4193 (3)	0.47162 (16)	0.09856 (18)	0.0577 (7)	
H10	0.3863	0.5209	0.1027	0.069*	
C11	0.5162 (2)	0.44323 (14)	0.16789 (15)	0.0419 (5)	
O12	0.70423 (17)	0.26675 (10)	0.21223 (11)	0.0547 (5)	
C13	0.8178 (3)	0.23953 (18)	0.26762 (17)	0.0567 (7)	
H13A	0.8795	0.281	0.2763	0.068*	
H13B	0.8507	0.197	0.2388	0.068*	
C14	0.8005 (3)	0.21225 (15)	0.35539 (18)	0.0524 (6)	
O15	0.91239 (17)	0.20395 (12)	0.40756 (12)	0.0601 (5)	
C16	0.9319 (3)	0.1729 (2)	0.49902 (19)	0.0689 (9)	
C17	1.0716 (6)	0.1585 (9)	0.5257 (5)	0.103 (3)	0.712 (18)
H17A	1.0937	0.1163	0.4921	0.154*	0.712 (18)
H17B	1.0941	0.1456	0.5868	0.154*	0.712 (18)
H17C	1.116	0.2045	0.5153	0.154*	0.712 (18)
C18	0.8890 (9)	0.2344 (8)	0.5518 (6)	0.102 (3)	0.712 (18)
H18A	0.8001	0.2423	0.5312	0.154*	0.712 (18)
H18B	0.9327	0.2819	0.5461	0.154*	0.712 (18)
H18C	0.9064	0.219	0.6121	0.154*	0.712 (18)
C19	0.8578 (12)	0.0967 (6)	0.5022 (6)	0.104 (3)	0.712 (18)
H19A	0.7691	0.107	0.4844	0.156*	0.712 (18)
H19B	0.8767	0.0766	0.5608	0.156*	0.712 (18)
H19C	0.8814	0.0593	0.4634	0.156*	0.712 (18)
C17A	1.0694 (16)	0.2040 (12)	0.5313 (14)	0.099 (6)	0.288 (18)
H17D	1.1222	0.1825	0.4958	0.149*	0.288 (18)
H17E	1.1009	0.1891	0.5912	0.149*	0.288 (18)
H17F	1.0694	0.2596	0.5268	0.149*	0.288 (18)
C18A	0.853 (3)	0.204 (2)	0.555 (2)	0.121 (9)	0.288 (18)
H18D	0.8686	0.2581	0.5645	0.181*	0.288 (18)
H18E	0.8715	0.177	0.6104	0.181*	0.288 (18)
H18F	0.7656	0.1958	0.5275	0.181*	0.288 (18)
C19A	0.931 (3)	0.0877 (13)	0.4806 (15)	0.111 (6)	0.288 (18)
H19D	0.9808	0.0773	0.4386	0.167*	0.288 (18)
H19E	0.8459	0.0709	0.4576	0.167*	0.288 (18)
H19F	0.965	0.0601	0.5338	0.167*	0.288 (18)
O20	0.7012 (2)	0.20044 (15)	0.37241 (16)	0.0819 (7)	
C21	0.6324 (2)	0.64392 (14)	0.23265 (15)	0.0429 (5)	
C22	0.7429 (2)	0.61663 (16)	0.21445 (17)	0.0506 (6)	
H22	0.7567	0.5636	0.2106	0.061*	
C23	0.8317 (3)	0.66926 (18)	0.2022 (2)	0.0638 (8)	
H23	0.906	0.652	0.1887	0.077*	
C24	0.8110 (3)	0.74818 (19)	0.2099 (2)	0.0663 (8)	
H24	0.8726	0.7836	0.2033	0.08*	
C25	0.6996 (3)	0.77424 (17)	0.2272 (2)	0.0627 (8)	
H25	0.6859	0.8272	0.2319	0.075*	
C26	0.6086 (3)	0.72214 (15)	0.23744 (18)	0.0541 (7)	
H26	0.5321	0.7394	0.2475	0.065*	
C27	0.5201 (2)	0.59711 (14)	0.36753 (16)	0.0445 (6)	
C28	0.6304 (3)	0.61895 (15)	0.42591 (18)	0.0526 (6)	
H28	0.7051	0.6245	0.407	0.063*	
C29	0.6276 (3)	0.63210 (18)	0.51133 (19)	0.0628 (8)	
H29	0.7004	0.6474	0.5509	0.075*	
C30	0.5158 (3)	0.6227 (2)	0.5391 (2)	0.0728 (9)	
H30	0.5143	0.6307	0.5975	0.087*	
C31	0.4080 (3)	0.6016 (2)	0.4807 (2)	0.0740 (9)	
H31	0.3334	0.5964	0.4997	0.089*	
C32	0.4086 (3)	0.58824 (17)	0.39439 (19)	0.0583 (7)	
H32	0.3353	0.5735	0.3549	0.07*	
S33	0.08006 (9)	0.48351 (5)	0.27300 (7)	0.0775 (3)	
O34	0.0972 (4)	0.56387 (19)	0.2559 (3)	0.1537 (16)	
O35	0.1986 (3)	0.44518 (19)	0.2888 (2)	0.1282 (12)	
O36	−0.0239 (4)	0.4482 (4)	0.2255 (3)	0.214 (3)	
C37	0.0488 (4)	0.4912 (3)	0.3778 (3)	0.0975 (13)	
F38	0.1375 (2)	0.5149 (2)	0.44055 (19)	0.1437 (12)	
F39	−0.0574 (2)	0.5243 (2)	0.3812 (2)	0.1429 (11)	
F40	0.0294 (4)	0.4167 (3)	0.4097 (2)	0.1917 (17)	

### Atomic displacement parameters (Å^2^)

**Table:**

	*U*^11^	*U*^22^	*U*^33^	*U*^12^	*U*^13^	*U*^23^
S1	0.0412 (3)	0.0396 (3)	0.0465 (4)	−0.0011 (2)	0.0040 (2)	0.0010 (3)
C2	0.0430 (13)	0.0356 (12)	0.0466 (13)	−0.0031 (10)	0.0065 (10)	0.0002 (10)
C3	0.0491 (14)	0.0419 (13)	0.0426 (13)	−0.0056 (11)	0.0014 (11)	0.0001 (11)
C4	0.0450 (13)	0.0427 (13)	0.0469 (14)	0.0015 (11)	−0.0030 (11)	0.0044 (11)
C5	0.0421 (12)	0.0378 (13)	0.0453 (13)	−0.0015 (10)	0.0079 (10)	0.0032 (10)
C6	0.0414 (12)	0.0416 (13)	0.0421 (13)	−0.0056 (10)	0.0066 (10)	0.0032 (10)
C7	0.0621 (16)	0.0488 (15)	0.0473 (15)	−0.0019 (12)	0.0031 (12)	−0.0049 (12)
C8	0.076 (2)	0.0631 (19)	0.0499 (16)	−0.0016 (16)	−0.0104 (14)	−0.0082 (14)
C9	0.0669 (19)	0.0662 (19)	0.0541 (17)	0.0062 (15)	−0.0157 (14)	−0.0019 (14)
C10	0.0559 (16)	0.0484 (15)	0.0584 (17)	0.0051 (12)	−0.0093 (13)	−0.0002 (12)
C11	0.0392 (12)	0.0417 (13)	0.0423 (13)	−0.0045 (10)	0.0035 (10)	0.0022 (10)
O12	0.0602 (11)	0.0473 (10)	0.0507 (10)	0.0109 (8)	−0.0002 (8)	−0.0016 (8)
C13	0.0539 (16)	0.0595 (17)	0.0544 (16)	0.0171 (13)	0.0073 (13)	0.0041 (13)
C14	0.0495 (15)	0.0458 (15)	0.0595 (17)	0.0053 (12)	0.0073 (12)	0.0034 (12)
O15	0.0485 (10)	0.0819 (14)	0.0478 (10)	0.0098 (9)	0.0060 (8)	0.0034 (9)
C16	0.0629 (19)	0.093 (2)	0.0461 (16)	0.0036 (17)	0.0016 (14)	0.0035 (16)
C17	0.068 (4)	0.159 (10)	0.074 (4)	0.037 (5)	0.003 (3)	0.040 (5)
C18	0.124 (7)	0.129 (9)	0.064 (4)	0.001 (5)	0.042 (4)	−0.015 (5)
C19	0.118 (7)	0.096 (5)	0.083 (5)	−0.001 (5)	−0.008 (4)	0.037 (4)
C17A	0.108 (12)	0.085	0.078 (9)	0.034 (9)	−0.039 (8)	0.006 (10)
C18A	0.147	0.13 (2)	0.110 (15)	0.041 (16)	0.085 (12)	0.053 (16)
C19A	0.148	0.082 (11)	0.084 (12)	0.018 (14)	−0.016 (13)	0.027 (9)
O20	0.0509 (12)	0.1047 (19)	0.0872 (16)	−0.0037 (12)	0.0089 (11)	0.0329 (14)
C21	0.0425 (12)	0.0428 (13)	0.0413 (13)	0.0002 (10)	0.0045 (10)	0.0051 (10)
C22	0.0498 (14)	0.0498 (15)	0.0515 (15)	0.0054 (12)	0.0101 (12)	0.0065 (12)
C23	0.0485 (15)	0.069 (2)	0.076 (2)	0.0074 (14)	0.0190 (14)	0.0216 (16)
C24	0.0543 (17)	0.0652 (19)	0.075 (2)	−0.0119 (15)	0.0048 (14)	0.0228 (16)
C25	0.0677 (19)	0.0427 (15)	0.076 (2)	−0.0025 (13)	0.0129 (16)	0.0092 (14)
C26	0.0519 (15)	0.0427 (14)	0.0671 (18)	0.0037 (12)	0.0119 (13)	0.0025 (13)
C27	0.0442 (13)	0.0413 (13)	0.0470 (14)	−0.0007 (10)	0.0081 (10)	−0.0010 (11)
C28	0.0483 (14)	0.0549 (16)	0.0528 (16)	0.0001 (12)	0.0072 (12)	−0.0024 (13)
C29	0.0693 (19)	0.0636 (19)	0.0516 (17)	−0.0022 (15)	0.0049 (14)	−0.0032 (14)
C30	0.090 (2)	0.077 (2)	0.0558 (18)	−0.0108 (18)	0.0252 (17)	−0.0032 (16)
C31	0.073 (2)	0.086 (2)	0.072 (2)	−0.0180 (18)	0.0340 (18)	−0.0019 (18)
C32	0.0518 (16)	0.0643 (18)	0.0603 (17)	−0.0156 (13)	0.0155 (13)	−0.0022 (14)
S33	0.0737 (6)	0.0603 (5)	0.0935 (7)	0.0028 (4)	0.0081 (5)	0.0009 (4)
O34	0.167 (4)	0.087 (2)	0.224 (4)	0.043 (2)	0.078 (3)	0.067 (2)
O35	0.131 (3)	0.111 (2)	0.144 (3)	0.054 (2)	0.033 (2)	−0.014 (2)
O36	0.162 (4)	0.366 (7)	0.116 (3)	−0.150 (4)	0.033 (2)	−0.112 (4)
C37	0.063 (2)	0.115 (3)	0.109 (3)	−0.013 (2)	0.007 (2)	−0.020 (3)
F38	0.0843 (17)	0.229 (4)	0.108 (2)	−0.0313 (19)	0.0021 (15)	−0.039 (2)
F39	0.0711 (16)	0.225 (4)	0.135 (2)	0.0091 (19)	0.0277 (15)	−0.024 (2)
F40	0.206 (4)	0.224 (4)	0.144 (3)	−0.043 (3)	0.036 (3)	0.059 (3)

### Geometric parameters (Å, º)

**Table:**

S1—C2	1.779 (2)	C19—H19B	0.96
S1—C27	1.783 (3)	C19—H19C	0.96
S1—C21	1.792 (2)	C17A—H17D	0.96
C2—C3	1.347 (3)	C17A—H17E	0.96
C2—C11	1.433 (3)	C17A—H17F	0.96
C3—C4	1.393 (4)	C18A—H18D	0.96
C3—H3	0.93	C18A—H18E	0.96
C4—C5	1.365 (3)	C18A—H18F	0.96
C4—H4	0.93	C19A—H19D	0.96
C5—O12	1.355 (3)	C19A—H19E	0.96
C5—C6	1.434 (3)	C19A—H19F	0.96
C6—C7	1.406 (3)	C21—C26	1.377 (3)
C6—C11	1.415 (3)	C21—C22	1.380 (4)
C7—C8	1.357 (4)	C22—C23	1.370 (4)
C7—H7	0.93	C22—H22	0.93
C8—C9	1.394 (4)	C23—C24	1.387 (4)
C8—H8	0.93	C23—H23	0.93
C9—C10	1.351 (4)	C24—C25	1.376 (4)
C9—H9	0.93	C24—H24	0.93
C10—C11	1.423 (3)	C25—C26	1.373 (4)
C10—H10	0.93	C25—H25	0.93
O12—C13	1.423 (3)	C26—H26	0.93
C13—C14	1.505 (4)	C27—C32	1.379 (4)
C13—H13A	0.97	C27—C28	1.391 (4)
C13—H13B	0.97	C28—C29	1.365 (4)
C14—O20	1.188 (3)	C28—H28	0.93
C14—O15	1.317 (3)	C29—C30	1.391 (5)
O15—C16	1.500 (3)	C29—H29	0.93
C16—C18A	1.46 (3)	C30—C31	1.368 (5)
C16—C18	1.482 (12)	C30—H30	0.93
C16—C19A	1.50 (2)	C31—C32	1.374 (4)
C16—C17	1.507 (7)	C31—H31	0.93
C16—C19	1.547 (10)	C32—H32	0.93
C16—C17A	1.565 (15)	S33—O36	1.354 (3)
C17—H17A	0.96	S33—O35	1.422 (3)
C17—H17B	0.96	S33—O34	1.429 (3)
C17—H17C	0.96	S33—C37	1.755 (5)
C18—H18A	0.96	C37—F38	1.282 (4)
C18—H18B	0.96	C37—F39	1.302 (5)
C18—H18C	0.96	C37—F40	1.409 (5)
C19—H19A	0.96		
			
C2—S1—C27	105.56 (11)	H19A—C19—H19B	109.5
C2—S1—C21	105.10 (11)	C16—C19—H19C	109.5
C27—S1—C21	102.43 (11)	H19A—C19—H19C	109.5
C3—C2—C11	122.0 (2)	H19B—C19—H19C	109.5
C3—C2—S1	121.34 (19)	C16—C17A—H17D	109.5
C11—C2—S1	116.53 (18)	C16—C17A—H17E	109.5
C2—C3—C4	120.6 (2)	H17D—C17A—H17E	109.5
C2—C3—H3	119.7	C16—C17A—H17F	109.5
C4—C3—H3	119.7	H17D—C17A—H17F	109.5
C5—C4—C3	120.3 (2)	H17E—C17A—H17F	109.5
C5—C4—H4	119.8	C16—C18A—H18D	109.5
C3—C4—H4	119.8	C16—C18A—H18E	109.5
O12—C5—C4	124.9 (2)	H18D—C18A—H18E	109.5
O12—C5—C6	114.4 (2)	C16—C18A—H18F	109.5
C4—C5—C6	120.8 (2)	H18D—C18A—H18F	109.5
C7—C6—C11	119.5 (2)	H18E—C18A—H18F	109.5
C7—C6—C5	121.7 (2)	C16—C19A—H19D	109.5
C11—C6—C5	118.9 (2)	C16—C19A—H19E	109.5
C8—C7—C6	120.6 (3)	H19D—C19A—H19E	109.5
C8—C7—H7	119.7	C16—C19A—H19F	109.5
C6—C7—H7	119.7	H19D—C19A—H19F	109.5
C7—C8—C9	120.4 (3)	H19E—C19A—H19F	109.5
C7—C8—H8	119.8	C26—C21—C22	121.9 (2)
C9—C8—H8	119.8	C26—C21—S1	115.1 (2)
C10—C9—C8	120.8 (3)	C22—C21—S1	123.0 (2)
C10—C9—H9	119.6	C23—C22—C21	118.7 (3)
C8—C9—H9	119.6	C23—C22—H22	120.7
C9—C10—C11	120.9 (3)	C21—C22—H22	120.7
C9—C10—H10	119.6	C22—C23—C24	120.2 (3)
C11—C10—H10	119.6	C22—C23—H23	119.9
C6—C11—C10	117.8 (2)	C24—C23—H23	119.9
C6—C11—C2	117.4 (2)	C25—C24—C23	120.3 (3)
C10—C11—C2	124.7 (2)	C25—C24—H24	119.9
C5—O12—C13	117.8 (2)	C23—C24—H24	119.9
O12—C13—C14	113.1 (2)	C26—C25—C24	120.1 (3)
O12—C13—H13A	109	C26—C25—H25	119.9
C14—C13—H13A	109	C24—C25—H25	119.9
O12—C13—H13B	109	C25—C26—C21	118.9 (3)
C14—C13—H13B	109	C25—C26—H26	120.6
H13A—C13—H13B	107.8	C21—C26—H26	120.6
O20—C14—O15	127.3 (3)	C32—C27—C28	121.3 (3)
O20—C14—C13	124.3 (3)	C32—C27—S1	115.6 (2)
O15—C14—C13	108.4 (2)	C28—C27—S1	123.0 (2)
C14—O15—C16	123.2 (2)	C29—C28—C27	119.0 (3)
C18A—C16—C19A	119.4 (14)	C29—C28—H28	120.5
C18A—C16—O15	118.0 (11)	C27—C28—H28	120.5
C18—C16—O15	106.4 (5)	C28—C29—C30	120.0 (3)
C19A—C16—O15	99.9 (10)	C28—C29—H29	120
C18—C16—C17	112.8 (6)	C30—C29—H29	120
O15—C16—C17	104.1 (4)	C31—C30—C29	120.2 (3)
C18—C16—C19	110.9 (5)	C31—C30—H30	119.9
O15—C16—C19	111.6 (4)	C29—C30—H30	119.9
C17—C16—C19	110.7 (5)	C30—C31—C32	120.8 (3)
C18A—C16—C17A	109.4 (16)	C30—C31—H31	119.6
C19A—C16—C17A	111.4 (11)	C32—C31—H31	119.6
O15—C16—C17A	96.3 (8)	C31—C32—C27	118.6 (3)
C16—C17—H17A	109.5	C31—C32—H32	120.7
C16—C17—H17B	109.5	C27—C32—H32	120.7
H17A—C17—H17B	109.5	O36—S33—O35	120.3 (3)
C16—C17—H17C	109.5	O36—S33—O34	117.4 (4)
H17A—C17—H17C	109.5	O35—S33—O34	109.3 (2)
H17B—C17—H17C	109.5	O36—S33—C37	103.7 (2)
C16—C18—H18A	109.5	O35—S33—C37	103.2 (2)
C16—C18—H18B	109.5	O34—S33—C37	99.4 (3)
H18A—C18—H18B	109.5	F38—C37—F39	110.7 (4)
C16—C18—H18C	109.5	F38—C37—F40	99.4 (4)
H18A—C18—H18C	109.5	F39—C37—F40	100.3 (4)
H18B—C18—H18C	109.5	F38—C37—S33	118.2 (3)
C16—C19—H19A	109.5	F39—C37—S33	115.5 (3)
C16—C19—H19B	109.5	F40—C37—S33	109.8 (3)
